# Occlusion, acid resistance, and elemental characterization of dentin treated with desensitizing agents

**DOI:** 10.1590/1807-3107bor-2025.vol39.016

**Published:** 2025-02-07

**Authors:** Maria Carolina Lopes de Souza RIBEIRO, Beatriz Araújo Jacinto FERREIRA, Ana Carolina Freitas RIBEIRO, Fabiana Mantovani Gomes FRANÇA, Cecilia Pedroso TURSSi, Roberta Tarkany BASTING, Waldemir Francisco VIEIRA-JUNIOR

**Affiliations:** (a) Faculdade São Leopoldo Mandic, School of Odontology, Campinas, SP, Brazil.; (b) Universidade Estadual de Campinas – Unicamp, Faculdade de Odontologia de Piracicaba, Piracicaba, SP, Brazil.

**Keywords:** Dentin Desensitizing Agents, Tooth Erosion, Dentin

## Abstract

The objective of this study was to evaluate the occlusion potential of in-office desensitizing agents, and characterize the human dentin elements after acid exposure. Twelve human dentin discs were sectioned into four specimens each, and randomized into treatments (n = 20): no treatment (negative control); no treatment and 6% citric acid exposure (positive control); application of Gluma desensitizer (Heraeus Kulzer) or PRG Barrier Coat (Shofu), followed by 6% citric acid exposure. Occlusion and dentin surface characteristics were determined by scanning electron microscopy (SEM, n = 10), and elemental composition (at%), by energy-dispersive X-ray spectroscopy (EDS, n = 10). Three calibrated, blinded evaluators used SEM to categorize the occlusion potential: 1 = occluded, 2 = partially unoccluded, 3 = equally occluded/unoccluded, 4 = partially occluded, 5 = unoccluded. Data were analyzed by weighted kappa, Friedman, and Nemenyi tests (α = 0.05). For SEM, mean occlusion scores were higher for the PRG Barrier Coat than the positive control (p = 0.0235). Most specimens in the controls scored 4 or 5. The most frequent scores for PRG Barrier Coat were 1(60%) and 2(20%), while 30% of Gluma specimens scored 1 and 2. Gluma showed intratubular precipitation, while PRG Barrier Coat covered dentinal tubules totally or partially. For EDS, the K% was lower for Gluma than the negative control (p = 0.0046), with Si peaks in dentin treated with PRG Barrier Coat. The bioactive in-office desensitizing agent with S-PRG filler (PRG Barrier Coat) promoted dentin tubule occlusion, and persisted after exposure to acid.

## Introduction

Dentin hypersensitivity (DH) is a clinically and scientifically relevant condition. In addition to being of multifactorial origin, it is characterized by acute stimulated pain of short duration.^1-3^ The most widely accepted mechanism for explaining the pain caused by DH is the hydrodynamic theory.^4,5^ This theory posits that stimuli evoked by exposed dentin cause an increase in fluid movement within the dentinal tubules, and that chemical, thermal, tactile, evaporative, or osmotic stimuli are some triggers of the nociceptor stimulus.^4^ However, other theories have also been put forth to explain the origin of the pain felt by the patient, such as the neural theory and the odontoblastic transduction theory.^6^

Some factors may promote a greater risk of developing DH, such as patients undergoing periodontal treatment, bulimics, those with salivary disorders, consumers of acidic foods and/or drinks, athletes, drug users, individuals who apply exaggerated force when toothbrushing, or those who have gastroesophageal diseases.^3,6,7^ Therefore, prevention, diagnosis, and the elimination of contributing factors are crucial for managing DH. However, many professionals feel inadequate to intervene effectively in cases involving this condition.^8^ DH is often associated with non-carious cervical lesions (NCCLs), which are located near the gingival margin. These legions are considered significant predisposing factors for DH, since they result in the loss of tooth structure, and subsequent dentin exposure.^9^ NCCLs have a complex multifactorial origin, and are characterized by the loss of dental tissue at the cementoenamel junction, resulting from mineral loss or wear that does not involve bacterial by-products.^9,10^

Some approaches and materials designed to control or treat DH have been tested and applied. They involve sealing the dentinal tubules or preventing the transmission of nociceptive impulses.^11^ Categorically, the agents can be subdivided into neural or occlusive actions.^6,7,12^ The mechanism of action of occlusion agents is to seal the dentinal tubules by mineral precipitation, protein precipitation, or remineralization of the structure. Sealing reduces the flow of fluid inside the tubule and dentin permeability.^13^ The ideal treatment to stop this pain would be a fast-acting, long-lasting, and painless agent, harmless to the pulp, and easy to apply.^6^ Different techniques and products have been suggested for in-office desensitizing treatment, and can be found in the form of gels, varnishes, solutions, sealants, ionomeric materials, and dentin adhesives, among others.^11^

Among the in-office desensitizing products, a solution containing glutaraldehyde and methacrylate (Gluma desensitizer, Heraeus Kulzer) is considered effective.^13^ It acts by occluding the dentinal tubules, and neutralizing the hydrodynamic movement mechanism.^14^ New bioactive materials, and products containing the S-PRG (surface pre-reacted glass) particle, have been suggested to treat DH.^15^ This particle has a pre-reacted layer of polyacrylic acid and silica, an ionic layer, and a multifunctional glass core.^16^ Although it has not been extensively validated for different clinical applications, the S-PRG particle can release ions stored in its ionic layer, such as sodium, aluminum, fluoride, borate, silicate, and strontium ions.^17,18^

The present study aimed to evaluate the in vitro performance of commercial products containing either glutaraldehyde and methacrylate (Gluma) or S-PRG filler (PRG Barrier Coat) by assessing their occlusion potential, and the elemental and mineral composition of human dentin under acid challenge. The following null hypotheses were tested: there are no significant differences a) between the treatments, concerning the elemental composition of the dentin, or b) between the groups, regarding the dentin occlusion pattern.

## Methods

### Experimental design

The study was an experimental, in vitro, randomized, and analysis-blinded investigation. The experimental units comprised twenty human dentin discs, each measuring 1.5 mm in thickness - ten designated for EDS, and ten for SEM. Each dentin disc was sectioned into four specimens randomly assigned to different treatment types. The specimen count was determined based on previous studies,^19^ with n = 10.

Treatments consisted of four levels:

- Negative control: untreated human dentin (distilled water);
- Positive control: untreated human dentin exposed to 6% citric acid;
- Dentin treated with a commercial product containing (2-hydroxyethyl) methacrylate and glutaraldehyde (Gluma desensitizer, Heraeus Kulzer, Wehreim, Germany) exposed to 6% citric acid;
- Dentin treated with a coating agent containing S-PRG filler (PRG Barrier Coat, Shofu, Japan) exposed to 6% citric acid.

Variables: % of elements (EDS) and occlusion scores (SEM) of human dentin, considering paired specimens.

### Specimens obtained

Healthy third molars extracted for orthodontic purposes were acquired after validation by the local ethics committee (CAAE: 61350322.9.0000.5374). Root planing and scaling were performed using periodontal curettes (Duflex, Rio de Janeiro, RJ, Brazil) to remove debris from the extracted teeth, which were then stored at 4°C under refrigeration until use. Third molars that were fractured, had enamel defects, carious lesions, or anatomical abnormalities were excluded from the study. Each tooth was affixed to acrylic plates and cut perpendicularly with a high-precision diamond blade (IsoMet 1000, Buehler, USA) to obtain 2 mm-thick portions. The teeth were sectioned 1.5 mm above the cementoenamel junction. A small mark was imprinted to identify the occlusal face. Next, both sides of the disc were sanded for 15 seconds with #600-grit sandpaper (3M, Sumaré, Brazil) to obtain surface uniformity and smoothness, and produce a 1.5 mm-thick specimen.

Two longitudinal cuts were made using a high-precision diamond blade (IsoMet 1000, Buehler) to divide the specimen into four equal parts.^22^ Each part was then randomized into a treatment group, allowing for final comparisons of composition and tubular characteristics with the same specimen and condition. Different discs were randomized for evaluation by SEM (n = 10) or EDS (n = 10), depending on the characteristics of each analysis. All the faces except the upper dentin/occlusal surface were protected with acid-resistant varnish (Colorama, Curitiba, Brazil). Next, the specimens were immersed in 17% EDTA solution for 5 minutes.^11,23^ The fragments were washed with distilled water, and placed in an ultrasonic cleaner (Marconi, Piracicaba, São Paulo, Brazil) for 10 minutes.

### Specimen treatment and citric acid exposure

The specimens were treated according to the manufacturer’s instructions. The composition of the materials is shown in Table 1. The following study groups were set up:

**Table 1:** Composition and products used in the experiment.

Product	Manufacturer	Composition^*^	Batch
Gluma desensitizer	Heraeus Kulzer	(2-hydroxyethyl) methacrylate (25-50%), glutaraldehyde	K010539
	GmbH, Wehreim, Germany	(5-10%), purified water	
PRG Barrier Coat	Shofu, Kyoto, Japan	S-PRG fillers, distilled water, methacrylic acid monomer, and other phosphonic acid monomers, BIS-MPEPP, carboxylic acid monomer, TEGDMA, initiator	11200

BIS-MPEPP: bisphenol A polyethoxy dimethacrylate; TEGDMA: triethylene glycol dimethacrylate; S-PRG: surface pre-reacted glass. *Manufacturer’s specifications or MSDS (Material Safety Data Sheet).

- Negative control: untreated human dentin (distilled water). Human dentin was not exposed to any treatment, and was immersed in distilled water for 5 minutes. Exposure to distilled water took place on a shaking table (SK 0330-Pro, Dragon Lab Laboratory Instruments, Beijing, China) at 100 rpm, as in the other groups exposed to 6% citric acid.
- Positive control: untreated human dentin exposed to 6% citric acid. Human dentin was not exposed to any treatment, and was immersed in 6% citric acid for 5 minutes,^24^ followed by washing with distilled water. Exposure to the acid took place on a shaking table at 100 rpm.
- Dentin treated with a commercial product containing (2-hydroxyethyl) methacrylate and glutaraldehyde (Gluma Desensitizer): the product was actively applied with a microbrush for 60 seconds on the established area, followed by application of an air jet, and abundant washing with water. After 24 hours of immersion in artificial saliva, the specimen was exposed to 6% citric acid for 5 minutes, as previously described.
- Dentin treated with a coating agent containing S-PRG filler (PRG Barrier Coat): the commercial material was applied for 3 seconds in a thin layer of the mixture on the previously dried tooth surface. The excess was then removed with a microbrush, and the specimen was light-cured for 10 seconds, using a light-curing device (Valo, Ultradent, Salt Lake City, UT, USA), in standard mode, with 1000 mW/cm^2^ of irradiance. After 24 hours of immersion in artificial saliva, the specimen was exposed to 6% citric acid for 5 minutes, as previously described.

The specimens were kept in a solution of artificial saliva for 24 hours before exposure to the treatments, and 24 hours after exposure to the acids. The artificial saliva composition was: methyl-p-hydroxybenzoate, 2·00 g/L; sodium carboxymethyl cellulose, 10·0 g/L; KCl, 0·625 g/L; MgCl_2_·6H_2_O, 0·059 g/L; CaCl_2_·2H_2_O, 0·166 g/L; K_2_HPO_4_, 0·804 g/L; KH_2_PO_4_, 0·326 g/L, with pH adjusted to 6.75 using KOH.^25,26^

### Scanning Electron Microscopy (SEM)

Lastly, the specimens underwent surface evaluation using SEM (Jeol, JSM 5600LV, Tokyo, Japan). Initially, the specimens were immersed in an ultrasonic cleaner (Marconi, Piracicaba, Brazil) for 10 minutes to remove non-adhered debris. The dentin specimens were prepared as previously described,^27^ and then rinsed with distilled water and dried in a desiccator. Each sample was affixed to a sample holder using double-sided carbon adhesive tape, coated with gold using a sputter coater (EMITCH, K450, UK), and then processed for imaging, followed by SEM analysis (TermoFisher Scientific, Quattro S model). The images were captured at 2000× magnification, focused on the central region of each specimen (voltage [kV]: 20; current: 32pA; spot size: 2.5).

Three blinded evaluators were selected and calibrated to qualify the tubular occlusion pattern taken from the images, based on an observer agreement ranging from substantial to almost perfect (95%), based on the weighted kappa index. The evaluators characterized the images according to the following scores: 1 = occluded (100%), 2 = partially unoccluded (50% to < 100% of occluded tubules), 3 = equally occluded/ unoccluded, 4 = partially occluded (<25% of occluded tubules), 5 = unoccluded.^28^

### Energy dispersive X-ray spectroscope (EDS)

The specimens were immersed in an ultrasonic tank (Marconi, Piracicaba, SP, Brazil) for 10 minutes to remove non-adhered debris, and were dried in a desiccator. EDS analysis was performed using SEM with an x-ray energy dispersive detector (Thermo Scientific UltraDry, model ANAX-60P-B, Brno, Czech Republic). Analysis characteristics: voltage (kV) = 20; current = 64pA; spot size = 3.0. A reading was taken at the central region of each sample at 1000× magnification for 60 seconds. EDS analysis was carried out on the dentin surface to determine the presence of elements such as Na, Mg, Al, Si, P, O, Cl, and Ca, among others.^29^ The atomic % (at%) was determined, and representative graphs were plotted.

### Statistical analysis

The inter-rater reproducibility analyses to establish the occlusion pattern were carried out to obtain the occlusion scores using the agreement percentages and weighted kappa, with 95% confidence intervals. Kappa was interpreted according to Landis and Koch.^30^ The result of the occlusion score was obtained from the mode of the three evaluators. Descriptive analyses were then conducted on the occlusion score data (absolute frequencies, relative frequencies, and quartiles), and the number of elements was quantified using EDS. The Friedman and Nemenyi tests were then used to compare the treatments, considering that each slice of dentin was sectioned and each subslice was exposed to a treatment. The analyses were performed using the R program (R Foundation for Statistical Computing, Vienna, Austria), with a significance level of 5%.

## Results

Table 2 presents the occlusion scores, with substantial agreement observed between the evaluators^30^ (evaluators 1 vs. 2: 75%, evaluators 1 vs. 3: 82.5%, evaluators 2 vs. 3: 67.5%) The weighted kappa confidence intervals were 0.65 (0.46–0.83), 0.77 (0.62–0.92) and 0.56 (0.35–0.76), respectively. Statistical analyses revealed significant differences in occlusion scores across the treatments (p = 0.0235). Tubule occlusion was significantly higher in the Barrier Coat-treated group compared with the untreated group exposed to citric acid (p = 0.0235), as indicated by the lower mean scores corresponding to increased tubule occlusion.

**Table 2:** Occlusion score results according to treatment.

Treatment	Minimum	First quartile	Median	Third quartile	Maximum	Mean
No treatment	2	3.2	4	4	5	2.8^ab^
No treatment/acid exposure	3	4	4	4	5	3.2^a^
Gluma/acid exposure	1	2.2	4	4	5	2.5^ab^
Barrier Coat/acid exposure	1	1	1	2	5	1.5^b^

Different letters indicate statistically significant differences (p ≤ 0.05). Friedman and Nemenyi test, p = 0.0235. Scores: 1 = occluded, 2 = partially unoccluded, 3 = equally occluded/ unoccluded, 4 = partially occluded, 5 = unoccluded.

**Figure 1:**
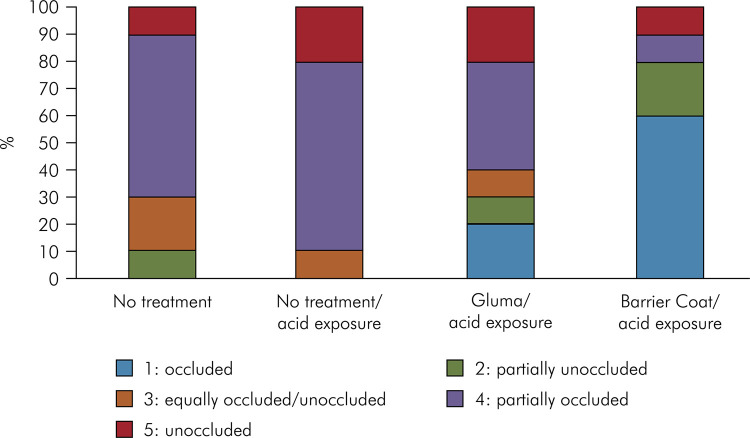
Distribution of the level of occlusion according to the groups.

Figure 1 shows the distribution (%) of scores according to the treatment. In the untreated/distilled water group, there was one sample (10%) classified as unoccluded, 60% of the specimens were partially occluded, and no blocks had an occluded score. Likewise, no blocks in the untreated/acid-exposed group had occluded or partially unoccluded scores. In the Barrier Coat-treated dentin group, 80% of the specimens were occluded or partially unoccluded, and 60% of these were completely occluded. All the occlusion patterns were observed in the group treated with Gluma, and 30% of the slices were classified as occluded or partially unoccluded.

**Figure 2:**
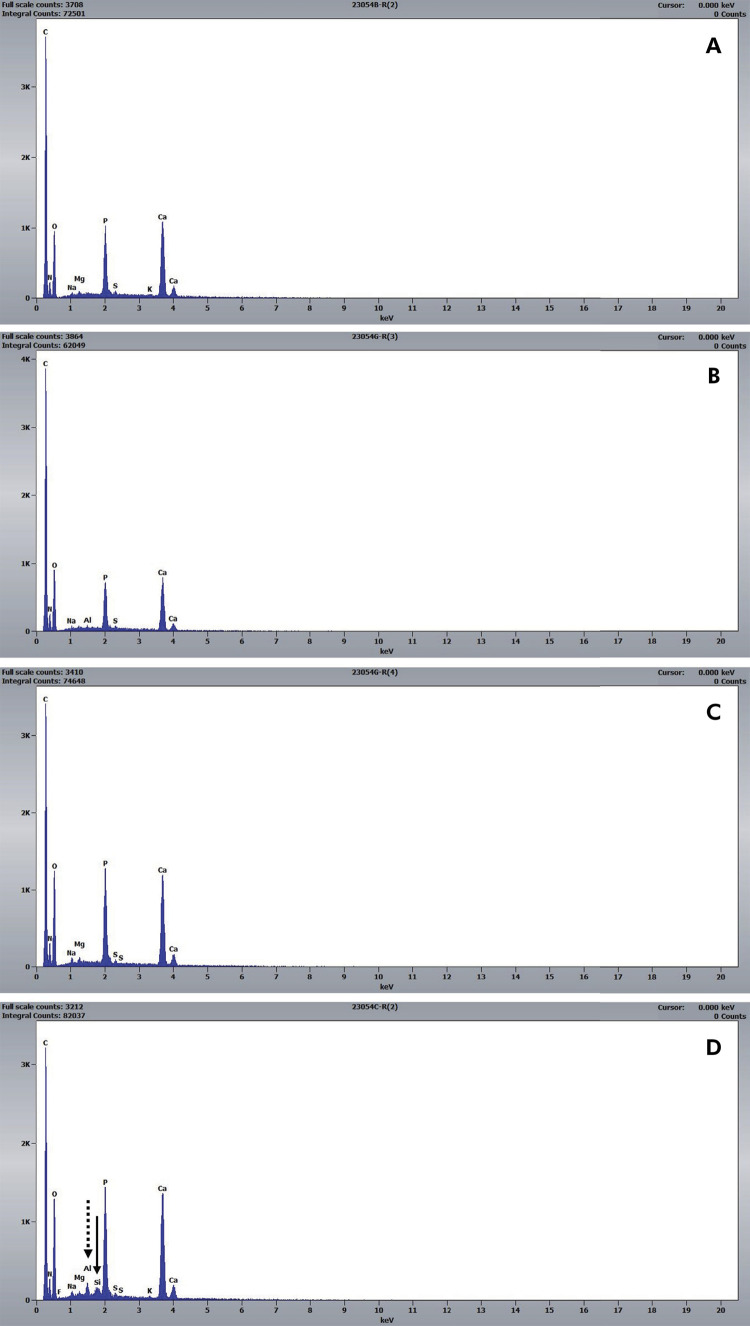
Representative graphs obtained by EDS.

The EDS analysis (Table 3 and Figure 2) identified the presence of Ca, K, Mg, C, N, Na, O, Cl, P, S, Si, and Al on the dentin surface. Table 3 indicates that the amount of K was significantly lower in the Gluma-treated dentin group, compared with the untreated/distilled water group (p < 0.05). Additionally, Si was detected in 5 out of 10 specimens within the group, although not in all the blocks evaluated (Table 3, Figure 2), while Al was found solely in the Barrier Coat-treated group (Figure 2). Cl was detected only in the untreated/distilled water and the Barrier Coat/acid-exposed dentin groups, as shown in Table 3.

**Table 3:** Median (minimum values; maximum values) of the elements (at%) according to treatment (n = 10).

Element	Treatment				p-value
	No treatment	No treatment/ acid exposure	Gluma/ acid exposure	Barrier Coat/ acid exposure	
C	44.6 (12.9; 54.5)^a^	36.3 (12.2; 47.5)^a^	35.4 (26.9; 47.9)^a^	42.1 (28.7; 58.3)^a^	0.17
O	37.9 (35.1; 58.6)^a^	35.8 (31.9; 59.7)^a^	36.5 (33.2; 44.6)^a^	38.1 (33.2; 44.6)^a^	0.37
N	19.5 (0; 22.1)^a^	22.2 (0; 23.9)^a^	19.2 (14.6; 23.4)^a^	15.5 (0; 24.0)^a^	0.27
Ca	5.5 (3.5; 18.2)^a^	5.1 (1.2; 16.8)^a^	4.2 (0.8; 19.7)^a^	3.8 (0.2; 9.0)^a^	0.51
P	2.9 (0.2; 10.6)^a^	3.45 (0.8;1 10.3)^a^	2.8 (0; 4.7)^a^	2.2 (0.6; 4.4)^a^	0.84
Na	0.3 (0.2; 0.9)^a^	0.2 (0; 0.8)^a^	0.3 (0; 0.4)^a^	0.2 (0; 0.35)^a^	0.26
Mg	0.2 (0.1; 0.5)^a^	0.2 (0; 0.3)^a^	0.2 (0.1; 0.3)^a^	0.1 (0; 0.2)^a^	0.49
K	0.04 (0; 0.26)^a^	0 (0; 0.05)^ab^	0 (0; 0.04)^b^	0.05 (0; 0.46)^ab^	0.01
S	0.05 (0; 0.12)^a^	0.06 (0; 0.11)^a^	0.05 (0; 0.11)^a^	0 (0; 0.09)^a^	0.77
Cl	0 (0; 0.1)*	0 (0; 0)	0 (0; 0)	0 (0; 0.04)^**^	-
Si	0 (0; 0)	0 (0;0)	0; (0; 0)	0 (0; 0.92)^***^	-

Distinct horizontal letters indicate statistically significant differences (p < 0.05). *presence of Cl in 40% of samples with mean = 0.03 and standard deviation = 0.04; ^**^presence of Cl in one sample. ^***^presence of Si in 50% of samples with mean = 0.21 and standard deviation = 0.34.

A: untreated/distilled water; B: untreated/acid exposure; C: gluma/acid exposure; D: Barrier Coat/Acid exposure. Arrows: D: Si and Al peaks.

**Figure 3:**
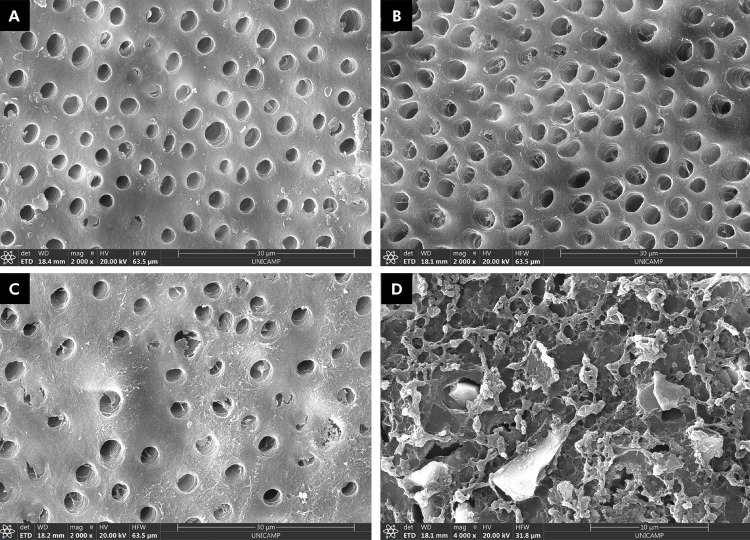
Images obtained at 2000× magnification.

The SEM images (Figure 3) demonstrate significant differences between the images of the control group (Figures 3A and 3B) and those of the treated group. The image of the untreated/distilled water group (Figure 3A) shows dentin with completely open dentinal tubules, characterizing an intact tubular pattern after treatment with a 17% EDTA solution. The untreated/acid-exposed dentin group (Figure 3B) displays a degraded surface, enlargement of the canaliculi openings, and the presence of fibrillar debris in the intratubular region. Conversely, the Gluma/acid-exposed dentin group (Figure 3C) suggests the preservation of peritubular dentin and intratubular precipitation, leading to partial tubule occlusion. In the Barrier Coat/acid-exposed group (Figure 3D), the dentinal tubules are completely covered by the material, indicating totally occluded dentin. Glassy particles of irregular sizes and shapes of the product can also be seen covering the dentin surface.

A: untreated/distilled water; B: untreated/acid exposure; C: gluma/acid exposure; D: Barrier Coat/Acid exposure.

## Discussion

DH can have an impact on the quality of life of affected patients.^2^ However, gaps remain in our understanding of the efficacy of DH treatments.^31^ This study aimed to investigate the in vitro performance of two commercially available products for DH treatment, focusing on their occlusion potential, and the elemental and mineral composition of dentin surfaces exposed to acid challenge. Based on the results, the null hypotheses were rejected, since there were statistically significant differences in both the occlusion patterns and the elemental characteristics of the dentin among the treatments tested.

Gels, solutions, varnishes, resin sealants, glass ionomers, and dentin adhesives have all been indicated as in-office treatments for DH to alleviate painful symptoms or achieve occlusion of dentin tubules.^3^ Tubular occlusion can be caused by the thickness of the dentin layer. Sensitive teeth exhibit a higher number of tubules (8×) and wider tubules (2×) in the buccal cervical area, compared with healthy teeth, along with increased fluid flow.^6,32^ Specimens were immersed in a 17% EDTA solution for 5 minutes to simulate conditions of DH, and remove the non-adhered smear layer, as evidenced by the SEM images. In addition, the dentin that was not exposed to any treatment and immersed in distilled water (negative control) presented a partially occluded (< 25% of occluded tubules, score 4) or unoccluded surface (score 5), characteristic of intact dentin exposed to EDTA.^11^ The specimens in the positive control were exposed to citric acid that had not been treated with any product. Acid exposure initially causes the outer layer of the demineralized matrix to suffer mineral loss and acid degradation, followed by detrimentally affecting a partially demineralized zone, and ultimately reaching more internal and solid portions of the dentin.^33,34^ In this sense, acid solutions can promote erosion of the peritubular dentin and removal of the smear layer (Figure 3), which is the main agent responsible for increased dentin permeability and likely clinical dentin hypersensitivity.^35^ In contrast, the groups treated with commercial products showed some occlusion potential.

The specimens exposed to Barrier Coat and immersed in acid were 60% totally occluded and 20% partially unoccluded, thus indicating that Barrier Coat achieved greater maintenance of tubular occlusion after acid exposure. This material presented an image with different characteristics from the untreated/acid-exposed group, where the dentinal tubules remained open and unoccluded. This can be explained by the composition of the material, namely, a light-cured resin matrix that remained on the dentin surface after acid exposure.^36^ PRG Barrier Coat light-curing varnish contains S-PRG particles formed by the acid-base reaction between aluminium fluoroborosilicate glass and polyacrylic acid, and promotes the release of ions.^36^ It also has resin monomers and carboxylic ions, which are needed for ionic bonding with the dentin substrate.^36^ This could explain why the material remained on the dentin after exposure to acid. In addition, EDS detected the presence of Si in this group, which is characteristic of glass, silicates, and silica particles common in materials containing S-PRG particles, because the outermost layer is covered with silicon oxide and/or is bioactive.

S-PRG filler can reduce mineral loss during acid challenges, and offer greater resistance to demineralization.^36^ PRG Barrier Coat is a light-cured material containing resin components, unlike Gluma, thus making it more effective at maintaining the varnish on the dentin surface. Although both contain resin monomers, PRG Barrier Coat includes phosphonic acid and methacrylic monomers, and is also photoactivated. Conversely, the specimens exposed to Gluma and immersed in acid showed only 20% occlusion, and 70% partial occlusion, as shown in the images, indicating a reduction in the dentinal tubule caliber with debris inside (Figure 3). Gluma desensitizer is an aqueous solution containing 5% glutaraldehyde and 35% hydroxyethyl methacrylate (HEMA). Glutaraldehyde occludes the dentinal tubules by coagulating amino acids and proteins present in the dentin, while HEMA acts by occluding the dentinal tubules.^37^ HEMA penetrates deeply into the dentinal tubules due to its hydrophilic nature. However, the blocking effect of HEMA is reversible, and the dentinal tubules become exposed after some time. Glutaraldehyde reacts with and coagulates the albumin serum present in dentin fluid,^38^ but the solutions used in this study did not contain albumin, and may have reduced the occlusion effects of Gluma. Various procedures can cause the loss of dentin fluid, including cutting, acid etching, immersion, and ultrasonic cleaning. When dentin is exposed, the fluid that comes out contains a fraction of plasma proteins; however, the levels in extracted teeth are lower than those of the teeth in the oral environment.^37^

The EDS analysis revealed the main component elements of dentin^27,39^ in all the test groups, as follows: C, O, P, and Ca. After acid exposure, Si (Table 3 and Figure 2) and Al (Figure 2) were also present in some specimens treated with Barrier Coat. This is because the material releases ions, and its particles are composed of aluminum fluoroborosilicate glass, which was maintained even after exposure to acid. Regarding the number of elements present in the specimens after exposure to acid, the only statistically significant difference between the groups was in regard to K, which was lower in the Gluma/acid-exposed group than the untreated control group. This can be explained by the mechanism of action of Gluma, and by the partial surface coverage with HEMA, which caused a reduction in the level of this element in the dentin.

This study used 6% citric acid to assess how well the products could resist the erosive challenges typical of the oral environment, thereby determining their acid resistance.^24^ As demonstrated in the present study, Mosquim et al.^40^ reported that PRG Barrier Coat resulted in more substantial deposits covering the dentin, and was more efficient following the erosive challenge. Additionally, another study^36^ indicated that PRG Barrier Coat can reduce dentin permeability even after erosive/abrasive challenges. Further research should validate the efficacy of these coating agents against various erosive and abrasive challenges, using different cycling models, and in the presence of salivary proteins. The limitations of the study are characteristic of in vitro models, particularly concerning the type of acid exposure, and the employment of qualitative and semi-quantitative methodologies. Moreover, alternative methodologies should be investigated to understand the mechanisms and longevity of mineral deposits and coating agent layers on dentin over time.

## Conclusion

The commercial products containing HEMA/glutaraldehyde (Gluma desensitizer) or S-PRG fillers (PRG Barrier Coat) were effective in promoting some tubule occlusion. Moreover, the coating agent with bioactive filler (PRG Barrier Coat) maintained the dentin covered after acid exposure. The products studied did not alter the composition of the human dentin elements.
